# Characteristics of the complete mitochondrial genome of *Gerres limbatus* (Cuvier, 1830) (Perciformes: Gerreidae)

**DOI:** 10.1080/23802359.2024.2333571

**Published:** 2024-04-02

**Authors:** Qi Zeng, Yu-jia Chen, Min Liu, Chen Wang

**Affiliations:** College of Ocean and Earth Sciences, Xiamen University, Xiamen City, Fujian Province, China

**Keywords:** *Gerres limbatus*, Gerreidae, Perciformes, mitochondrial genome, phylogenetic analysis

## Abstract

The saddleback silver-biddy *Gerres limbatus* (Cuvier 1830) is distributed in Indo-West Pacific Oceans and associated with shallow coastal marine waters and estuaries. In this study, the complete mitochondrial genome of *G. limbatus* was firstly documented, which is 16,730 bp in length, including 13 protein-coding genes, 22 transfer RNA genes and 2 ribosomal RNA genes. The overall base composition of the mitochondrial genome is 26.42% A, 28.68% C, 27.32% T, and 17.58% G. The Maximum Likelihood phylogenetic tree was constructed based on *COI* gene of the 31 species from the family Gerreidae, with *Heteroclinus puellarum* and *Hypopterus macropterus* as outgroups. It revealed that *G. erythrourus* was placed as the sister group to *G. limbatus.*

## Introduction

Genus *Gerres* is the largest in the eight genera of the family Gerreidae (Perciformes) and consists of 28 species (Nelson et al. 2016). Among these, 10 *Gerres* species are distributed in Chinese waters and are small to medium body sizes (fishdb.sinica.edu.tw, accessed 14 August 2023). *Gerres* species mainly feed on benthic animals, acting as low trophic level predators and transferring energy to higher trophic level predators (Chollet-Villalpando et al. 2014). Studies on the genus *Gerres* mainly focused on species taxonomy (Iwatsuki and Kimura 1998; Chollet-Villalpando et al. 2014).

The saddleback silver-biddy *Gerres limbatus* (Cuvier 1830) has a maximum of 15 cm in total length, and is mainly distributed in Indo-West Pacific Oceans, associated with shallow coastal marine waters and estuaries (www.fishbase.org, accessed 14 August 2023). In China, *G. limbatus* is common in the South China Sea and East China Sea (Liu et al. 2016).

The fish mitochondrial genome is a circular double-stranded DNA molecule, which has maternal inheritance, high copy number, high evolutionary rate and highly conserved (Boore 1999). To date, only four complete mitochondrial genomic sequences from the genus *Gerres* have been published (Ruan et al. 2020), including *G. decacanthus*, *G. erythrourus*, *G. filamentosus* and *G. oyena*. To fill in the data gaps at the molecular level, the complete mitochondrial genome of *G. limbatus* was sequenced.

## Materials and methods

**Figure 1.**
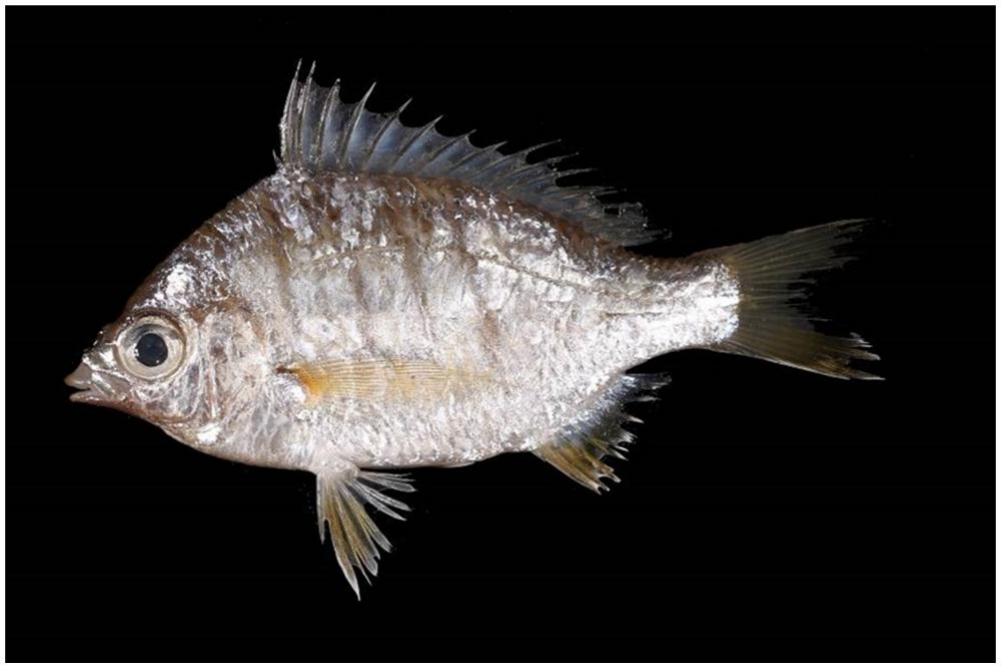
The saddleback silver-biddy *Gerres limbatus*.

A fresh specimen of *G. limbatus* (Figure 1) was collected by a bottom trawler in April 2022 in Xiamen Bay, Fujian Province (24°30′14″N, 118°17′27″E). It was stored at −20 °C in the Fish Biology Laboratory of Xiamen University (Dr. Chen Wang, wangchen2971@163.com) under the voucher number XM2023023. Genomic DNA was extracted from the gill tissue using the Marine Animal Tissue Genomic DNA Extraction Kit (DP324, Tiangen, China). The Ex Taq™ Version 2.0 plus dye (Takara) was employed to amplify sequence by polymerase chain reaction (PCR). Parameters of PCR reaction were set according to the instructions. The PCR primers were provided by the Supplementary Table 1.

Sequence data were analyzed and compiled using DNAStar (Burland 2000). The base composition was calculated by MEGAX (Kumar et al. 2018). The mitochondrial genes of *G. limbatus* were annotated by MITOS2 Web Server (http://mitos2.bioinf.uni-leipzig.de/index.py), and its mitochondrial genome map was produced using Proksee (https://proksee.ca/). To validate the phylogenetic position of *G. limbatus*, the phylogenetic tree was constructed using *COI* gene by the Maximum Likelihood (ML) method in IQ-Tree v1.6.2 (Minh et al. 2020), with 1,000,000 ultrafast bootstraps. It contains *G. limbatus* and other 30 species from the family Gerreidae, with *Heteroclinus puellarum* (HM902454) and *Hypopterus macropterus* (LC269833) as outgroups. The phylogenetic trees dataset files were used to visualize and annotate the phylograms in iTOL v6 (Letunic and Bork 2016).

## Results and discussion

**Figure 2.**
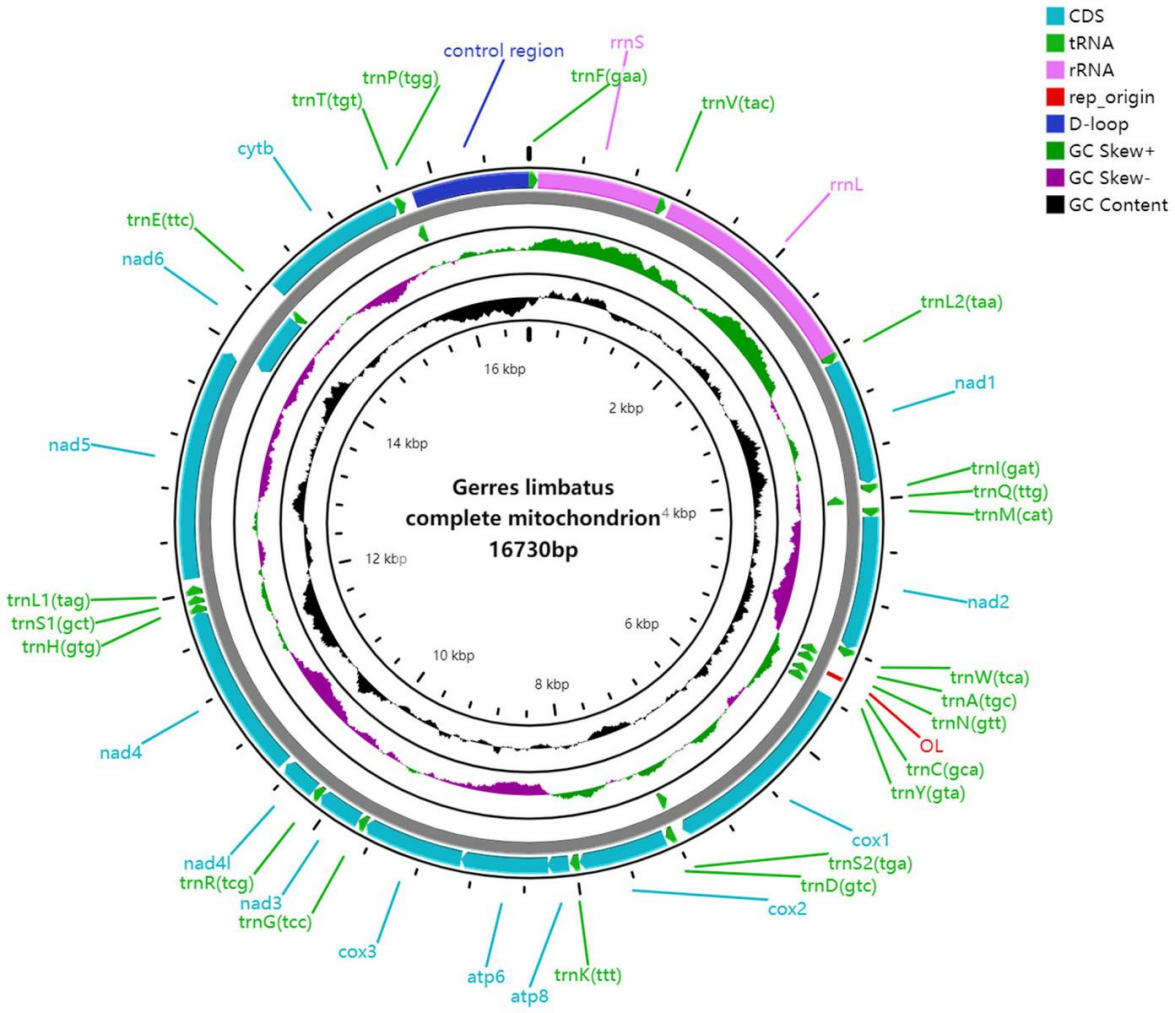
Graphic representation of the mitochondrial genome of *Gerres limbatus.*

The length of the complete mitochondrial genome for *G. limbatus* was 16,730 bp (Accession number: OR002186), including 13 protein-coding genes (PCGs), 22 transfer RNA (tRNA) genes, and 2 ribosomal RNA (rRNA) genes (Table 1; Figure 2).

**Table 1.** Features of the complete mitochondrial genome for *Gerres limbatus.*

Genes	Strand	Start/Stop	Size (bp)	**Intergenic Spacer** * **(bp)**	Codon Start/Stop	Anti-codon
tRNA-*Phe*	H	1/70	70	0		GAA
12S rRNA	H	71/1023	953	0		
tRNA-*Va*l	H	1024/1094	71	25		UAC
16S rRNA	H	1120/2826	1707	0		
tRNA-*Leu*	H	2827/2900	74	0		UAA
*ND1*	H	2901/3875	975	6	ATG/TAA	
tRNA-*Ile*	H	3882/3952	71	5		GAU
tRNA-*Gln*	L	3958/4028	71	37		UUG
tRNA-*Met*	H	4066/4134	69	0		CAU
*ND2*	H	4135/5181	1047	−1	ATG/TAA	
tRNA-*Trp*	H	5181/5252	72	1		UCA
tRNA-*Ala*	L	5254/5322	69	2		UGC
tRNA-*Asn*	L	5325/5397	73	5		GUU
O_L_	–	5403/5435	33	−1		
tRNA-*Cys*	L	5435/5502	68	0		GCA
tRNA-*Tyr*	L	5503/5574	72	1		GUA
*CO*I	H	5576/7128	1553	0	GTG/TAA	
tRNA-*Ser*	L	7129/7199	71	3		UGA
tRNA-*Asp*	H	7203/7274	72	10		GUC
*CO*II	H	7285/7975	691	0	ATG/T	
tRNA-*Lys*	H	7976/8050	75	8		UUU
*ATP8*	H	8059/8226	168	−7	ATG/TAA	
*ATP6*	H	8220/8900	681	−1	ATA/TAA	
*CO*III	H	8900/9685	786	0	ATG/TAA	
tRNA-*Gly*	H	9686/9755	70	0		UCC
*ND3*	H	9756/10104	349	0	ATG/T	
tRNA-*Arg*	H	10105/10173	69	0		UCG
*ND4L*	H	10174/10470	297	20	ATG/TAA	
*ND4*	H	10491/11856	1366	−12	ATG/T	
tRNA-*His*	H	11845/11913	69	0		GUG
tRNA-*Ser*	H	11914/11981	68	5		GCU
tRNA-*Leu*	H	11987/12060	74	52		UAG
*ND5*	H	12113/13927	1815	−15	ATG/TAA	
*ND6*	L	13913/14434	522	0	ATG/TAA	
tRNA-*Glu*	L	14435/14504	70	30		UUC
*Cytb*	H	14535/15675	1141	0	ATG/T	
tRNA-*Thr*	H	15676/15748	73	1		UGU
tRNA-*Pro*	L	15750/15821	72	139		UGG
D-loop	H	15822/16728	906	0		

* Positive values indicate distances between adjacent genes, negative values indicate the overlapping sequence of between adjacent genes.

The base composition of the *G. limbatus* mitogenome is 26.42% A, 28.68% C, 27.32% T, and 17.58% G, with a slightly AT-rich feature (53.74%). Eight tRNA genes (tRNA-*Ala*, tRNA-*Asn*, tRNA-*Cys*, tRNA-*Gln*, tRNA-*Glu*, tRNA-*Pro*, tRNA-*Ser* (TGA), and tRNA-*Tyr*) and *ND6* gene are encoded on the light chain, and other genes are encoded on the heavy chain. It has one control region, between tRNA-*Pro* gene and tRNA-*Phe* gene. The mitochondrial genome contains six overlapping regions and 18 intergenic regions. Most of the PCGs initiation codon is ATG, except *COI* gene starts with GTG. For the termination codons, nine PCGs (*ATP6*, *ATP8*, *COI*, *COIII*, *ND1*, *ND2*, *ND4L*, *ND5* and *ND6*) are terminated with the complete stop codon TAA, and the others (*COII*, *Cytb*, *ND3*, and *ND4*) are ended with the incomplete stop codon T.

**Figure 3.**
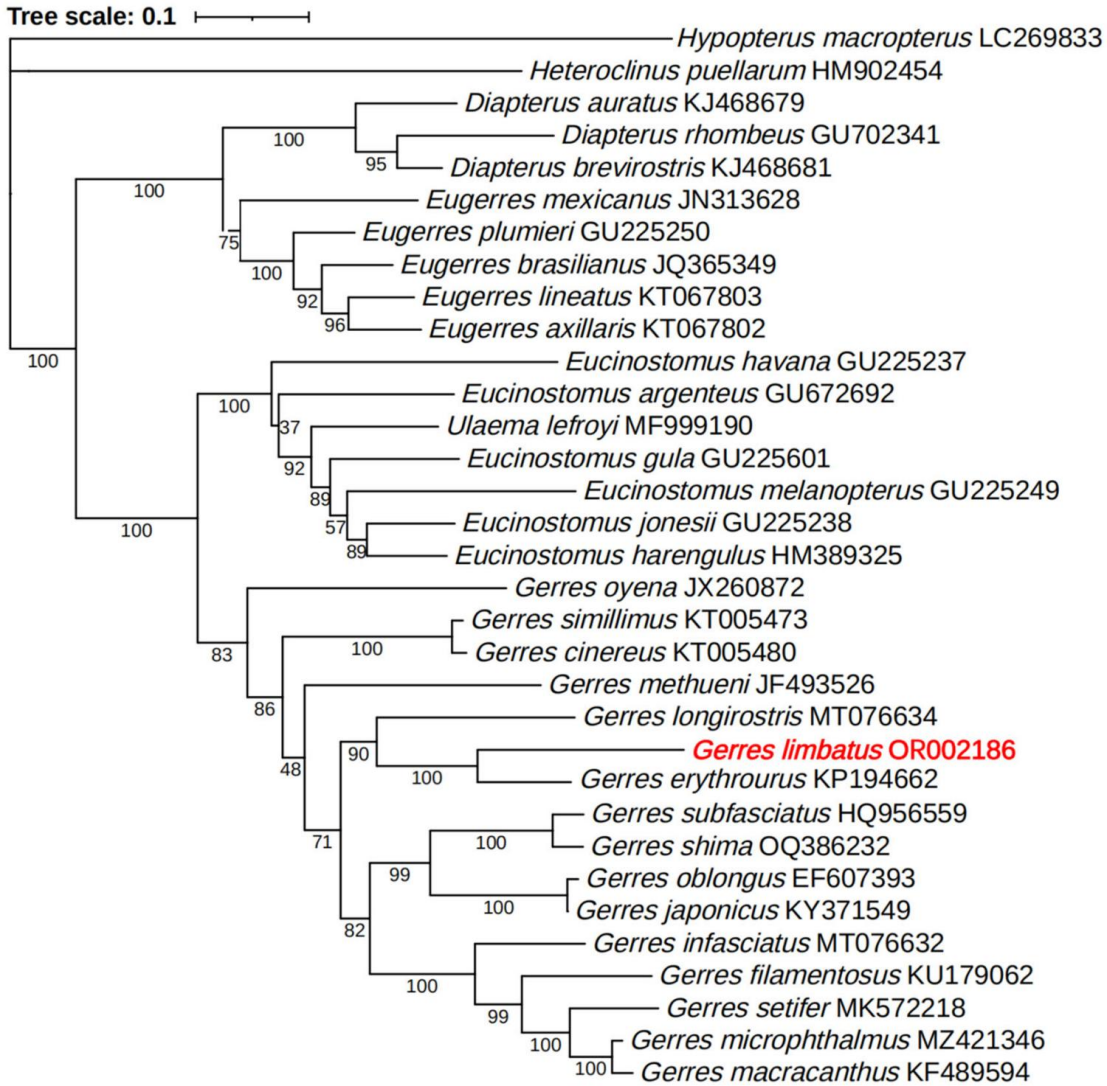
The phylogenetic tree of 31 species from the family gerreidae based on the *COI* gene was constructed with Maximum Likelihood method by IQ-tree. The following sequences were used: *Hypopterus macropterus* (LC269833, Iwatsuki et al. 2018), *Heteroclinus puellarum* (HM902454), *Diapterus auratus* (KJ468679, Vergara-Solana et al. 2014), *Diapterus rhombeus* (GU702341, Ribeiro et al. 2012), *Diapterus brevirostris* (KJ468681, Vergara-Solana et al. 2014), *Eugerres mexicanus* (JN313628), *Eugerres plumieri* (GU225250), *Eugerres brasilianus* (JQ365349, Ribeiro et al. 2012), *Eugerres lineatus* (KT067803), *Eugerres axillaris* (KT067802), *Eucinostomus havana* (GU225237), *Eucinostomus argenteus* (GU672692), *Ulaema lefroyi* (MF999190), *Eucinostomus gula* (GU225601), *Eucinostomus melanopterus* (GU225249), *Eucinostomus jonesii* (GU225238), *Eucinostomus harengulus* (HM389325), *Gerres oyena* (JX260872), *Gerres simillimus* (KT005473), *Gerres cinereus* (KT005480), *Gerres methueni* (JF493526), *Gerres longirostris* (MT076634), *Gerres erythrourus* (KP194662), *Gerres subfasciatus* (HQ956559), *Gerres shima* (OQ386232, Bemis et al. 2023), *Gerres oblongus* (EF607393, Zhang 2011), *Gerres japonicus* (KY371549), *Gerres infasciatus* (MT076632), *Gerres filamentosus* (KU179062), *Gerres setifer* (MK572218, Rahman et al. 2019), *Gerres microphthalmus* (MZ421346), *Gerres macracanthus* (KF489594).)

In the ML phylogenetic tree (Figure 3), the 16 *Gerres* species clustered a monophyletic group, located at the top of the Gerreidae phylogenetic tree with high support value. *Gerres erythrourus* clustered to *G. limbatus* as a sister group (bootstrap value = 100), then clustered to *G. longirostris* and other *Gerres* species.

## Discussion and conclusion

In this study, the complete mitochondrial genome of *G. limbatus* was firstly reported. The circle mitochondrial genome was 16,730 bp in length, containing 37 genes, including 13 PCGs, 22 tRNAs and two rRNAs. The results show that *G. limbatus* is closely related to *G. erythrourus*. The mitochondrial genomic data of *G. limbatus* will be useful for studies on species identification, biodiversity monitoring and conservation, DNA barcoding and population genetics in the family Gerreidae.

## Supplementary Material

Supplemental Material

## Data Availability

The mitochondrial genome sequence is available on GenBank of NCBI at (https://www.ncbi.nlm.nih.gov/) with the accession number of OR002186. The associated BioProject, BioSample and SRA numbers are PRJNA1025369 and SAMN37722177: XM2023023 (TaxID: 435152). We used the sanger method to obtain data without SRA number.
