# Supplementary material for: Characteristics of the complete mitochondrial genome of Gerres limbatus (Cuvier, 1830) (Perciformes: Gerreidae)
Source: Mitochondrial DNA B Resour. 2024 Apr 2;9(4):419–22. doi: 10.1080/23802359.2024.2333571 (PMC10993760; doi:10.1080/23802359.2024.2333571)
Supplement: Supplemental Material [file TMDN_A_2333571_SM0334.docx]

Supplementary table1. Eight primer pair sequences for mitogenomic amplification.

| **Primer** | **Sequence (5’-3’)** |
| --- | --- |
| 228EF | CTTGCTTWGCCACACCC |
| 2492EF | ACCCYAGGGATAACAGCG |
| 5131EF | AGACCAAGRGCCTTCAAAG |
| 7106EF | CCGCTCTGYCACTTTCTT |
| 9172EF | GACCYCCNACTGGSATTAC |
| 11774EF | CAAAAACATTAGATTGTGRTTC |
| 13158EF | AAAGAYGCYATYATTGAAGC |
| 15417EF | GGMATACCWGTAGAACACCC |
| 2674ER | TCTTTTCGGTCCTTTCGT |
| 5447ER | TTTAAGCGGTGGATTGTA |
| 7214ER | DGGKGAAGYTGCATCTTG |
| 9603ER | CTAGGTGATTGGAAGTCAC |
| 11937ER | RCYACTTGGAKTTGCACC |
| 14469ER | GGGARCCRAARTTTCATCA |
| 15554ER | CVTYCGGTTTACAAGACC |
| 489ER | GGGGTATCTAATCCCAGTTT |
